# Individual and environmental parameters in children with and without developmental coordination disorder: associations with physical activity and body mass index

**DOI:** 10.3389/fped.2023.1202488

**Published:** 2023-10-19

**Authors:** Nadia Cristina Valentini, Priscila Silva de Souza, Mariele Santayana de Souza, Glauber Carvalho Nobre

**Affiliations:** ^1^ School of Physical Education, Federal University of Rio Grande do Sul, Porto Alegre, Brazil; ^2^ Department of Physical Education and Sports, Federal University of Rio Grande do Sul, Porto Alegre, Brazil; ^3^ Department of Physical Education and Sports, Federal Institute of Education, Science and Technology of Ceará, Fortaleza, Brazil

**Keywords:** childhood, motor development disorders, motor performance, self-perceived abilities, physical education

## Abstract

**Introduction:**

Physical Activity (PA) is a complex behavior, and the relevance of other factors such as BMI, SES and children’s behavior at school and home still lacks investigation for children, especially those at risk or with Developmental Coordination Disorder (DCD). The objective of this study was to examine whether socioeconomic status (SES), school's spaces for children’s movement, active play, screen time, motor skill proficiency, perceived motor competence, and engagement in the physical education lessons were associated with PA and BMI in children with typical development (TD), at risk of DCD (r-DCD), and with DCD.

**Methods:**

Children (*N* = 352; 4–10-year-old) from six public schools in a major urban city, in southern of Brazil, were assessed regarding motor skill proficiency (locomotor and ball skills), perceived motor competence, and weight status. PA and engagement in the lesson were assessed using pedometers and a behavior checklist of motor experience of success. Parents recorded the daily time that children spent on screen and in active play.

**Results:**

The hierarchical multivariate linear regressions showed that age, sex, SES, Schools with more favorable space for children’s movement, locomotor and ball skills, and successful engagement, were associated with PA for children with TD. Age, screen time, locomotor, and successful engagement were associated with BMI. For children at r-DCD, age, sex, SES, with more favorable space for children’s movement, and locomotor were associated with PA. Age, active play and screen time were associated with BMI. For children with DCD, sex, SES, Schools with more favorable space for children’s movement, screen time, and successful engagement were associated with PA. Age, sex, active play, and screen time were associated with BMI.

**Conclusion:**

Different factors were associated with PA and BMI for children with different levels of motor impairment (r-DCD and DCD) and children with TD.

## Introduction

Worldwide, approximately 81% of young people aged 11–17 years were insufficiently physically active, with 85% of girls and 78% of boys not meeting the recommended minimum of 60 min of moderate- to vigorous-intensity physical activity (PA) per day (1). Recently, the Global Matrix of Report Cards (2), a study enrolling 49 countries from six continents, showed that around the world, only 30% of children are meeting the global recommendations on PA for health; for Brazilian children, the study reported an indicator of 34% to 39% (2). Furthermore, a recent systematic review showed that for Brazilian schoolchildren (6–17-year-olds), 27%–30% are in the health risk zone for Body Mass Index (BMI), 70% for cardiorespiratory fitness, and 50% and 65% for flexibility and muscular strength, respectively (3). A high prevalence of overweight (± 11%) and obesity (± 8%) has also been reported among Brazilian children (4).

Several factors are associated with PA and BMI, such as motor skill proficiency (MSP) (5) and perceived motor competence (6, 7). PA and weight status are determined by several biological, psychological, sociocultural, and environmental factors (8); therefore, these inconsistencies in the relationship and the controversial results are somewhat recurring. Previous systematic reviews showed the complexity of identifying correlates associated with PA in children. Eight systematic reviews showed heterogeneous and inconsistent results for several factors and consistent correlates were also identified. For example, children’s PA was reported to be positively associated with self-efficacy (9, 10), motor performance (11, 12), outdoor time (13), sex (for boys), and families' socioeconomic status (10). Most of the studies within these systematic reviews were conducted on children from rich and developed countries and research in low-income countries is scarce.

PA and weight status are complex and multidimensional factors; how they are affected during childhood by socioeconomic status (SES) and children’s behavior at school (i.e., engagement in lessons) and at home (i.e., active play and screen time) still lack investigation among children from low-income families. Children’s behavior is the product of changing relations and events between and the opportunities in the contexts. For example, screen time is related to less active behavior and a higher prevalence of overweight in children (14). Furthermore, PA behavior is strongly associated with locomotor skills proficiency for younger children, whereas, for older children, ball skills play a more vital role in PA engagement (15). If these categories of skills are differently associated with PA and BMI for children with a wide range of motor impairments, it has not yet been examined to the best of the author’s knowledge.

Moreover, high quality of engagement in PE lessons is strongly associated with children's PA (16). It is crucial to notice that modest evidence regarding children’s qualitative behavior in lessons is available. Nevertheless, for children with Development Coordination Disorder (DCD), successful motor experiences and whether their success is related to PA have not yet been addressed. Examining a child’s behavior is crucial to advancing comprehension of qualitative factors guiding children’s PA participation.

Furthermore, although considerable work addressing these factors has been published for children with typical development, much less is known about children with DCD. Children with DCD have difficulties in gross motor skills (17), less engagement in PA (18), the propensity to be overweight and obese (19); (20) as higher BMIs have been reported across ten cohort studies (21), and fragile self-perceptions (22, 23). Children with DCD with higher BMIs also demonstrate lower PA levels (19, 24). The higher the motor impairments, the lower the levels of physical activity and involvement in physical activities that could prevent health problems.

It is noteworthy that studies commonly investigate the different factors that affect children with DCD in isolation, but there is a lack of studies that pursue understanding how all these factors are interrelated and affect PA and BMI for children with a wide range of motor proficiency. Understanding those relationships is essential to developing appropriate strategies that promote children’s physical health and development. Therefore, the objective of this study is to examine whether perceived motor competence, MSP (locomotor and ball skills), SES, active playtime at home, screen time at home, school context, and successful motor engagement in physical education lessons are associated with PA and BMI in children from low-income families with typical development, at risk of DCD (r-DCD), and with DCD.

## Methods

### Participants and context

In this mixed-design study, initially, 360 children agreed to participate, but 8 discontinued their participation in the second week of the assessment and were excluded from the study. Therefore, 352 Children (49.7% girls), aged 4 to 10 years, from six public schools in a major urban city (nearly 1.5 million inhabitants) in southern Brazil participated in the study. The children’s daily outdoor routine included walking or riding bicycles to school and to meet friends near their house, playing games involving balls, jumping ropes, hide and seek, and tag games. Inside the home, most children were involved in helping with household chores, studying, doing homework, drawing, and watching TV; less than 20% of the sample also danced at home. Children also used the computer, but it was restricted to nearly 40% of the sample, and they shared it with parents and other brothers and sisters.

All children attended schools close to their homes; all schools were in peripheric regions, had outside courts for physical education lessons, and restricted equipment for physical education lessons. Some schools were located in high-density areas, and others had less physical space available for children's free play and physical education lessons; equipment availability was similar across all schools. Children were from low-income families composing, according to the Brazilian SES indicators (25), the socioeconomic groups “D” (monthly ± 2000 reals/ 400 dollars) and “C"(monthly income ± 4,000 reals /800 dollars). Many of the children in the present study (59.4%) lived at or below Brazil’s federal poverty line (Group “D”), all the families were enrolled in the governmental social programs receiving governmental support, and both parents worked. However, most jobs were low paying (± 80%), with no qualifications required and high turnover; around 20% of the parents had informal jobs. The families from group “C” lived in the same neighborhood; however, most parents finished high school and had more stay jobs. Human subject approval was obtained from the university ethical committee. Consent was obtained from each child’s custodial caregiver(s), and each child verbally agreed to participate in the study.

### Screening procedures for groups’ composition and sample size power by groups

First, we assessed the 352 children using the Movement Assessment Battery for Children—second edition (MABC-2) (26); adopting the recommended guidelines (27), children with scores at or below the fifth percentile should be considered unequivocal evidence of DCD (Criterion A), provided the child meets all other criteria. Parents' school reports were used to assess whether motor delays meaningfully interfered with the children’s daily activities at home, and the classroom teachers provided information regarding overall motor difficulties during school tasks (Criterion B). Parents provided total access to the child’s medical information in the school archives regarding intellectual capability to ensure that the motor skills deficits were not better explained by intellectual disability or not attributable to neurological or sensory conditions affecting movement (Criterion D) and regarding children’s motor milestone acquisition (Criterion C). Children with medical reports of disabilities and any neurological condition cases were excluded from the present study.

Consequently, from our initial sample, 83 (23.6%) children who scored at or below the fifth percentile (27) and met DSM V criteria [American Psychological Association (APA), 2013] comprised the DCD group. From the initial sample (*n* = 352), we also identified 98 (27.8%) children who scored between the 6th and the 15th percentile on MABC-2; those children showed subtle signs of motor impairments compared to those with DCD. Since they performed above the 5th percentile in the MABC-2 and did not have delays in motor milestone acquisition (criteria C), they composed the r-DCD group, as per previous studies (28, 29, 30). Furthermore, 171 (48.6) children scored above the 15th percentile on MABC-2 and were in the Typical Development group.

The posterior power (1—β error probability) of the hierarchical linear multiple regression models was conducted for sample size (n) for each group, the effect size (f2) observed in the regression analyses, the alpha error probability (α), and the number of factors (np). Thus, considering the *n* of the groups of children (TD = 172, r-DCD = 98 and DCD = 83), the f2 observed in the models an α of <.05, and an np = 10; the values of power observed in the models were between.82 and 1. Only 1 model showed a power value = .34.

### Assessment

#### Physical activity

Pedometers (Yamax Digiwalker SW-200; Yamasa, Tokyo, Japan) were used in the study, as in previous studies (31, 16). Pedometers are low in cost, their accuracy has been acknowledged, and they are an alternative to accelerometers as an objective measure of PA for large-scale research (32). Specifically, the results obtained from Yamax Digiwalker pedometers are reported as valid and reliable (33). Pedometers were placed at the children’s waistline (approximately at the midline of the thigh) in four physical education lessons with a duration of 45 min. Each participant’s total number of steps during each lesson was recorded, and an individual mean was obtained; the standard mean steps per minute were adopted as the PA measure. The examiners were responsible for testing all pedometers before each session and positioning the pedometers. The examiners asked the children to stand still in line and placed the pedometers on the children’s waists 10 min before the lessons started.

#### Body mass index

A trained professional conducted all the assessments. Children were asked to remove their shoes, and their height and weight were measured. Weight was measured using an electronically calibrated scale and recorded to the nearest 100 g. A portable stadiometer was used to measure standing height, with the value recorded to the nearest half-centimeter. BMI was determined (weight [kg] divided by height [m] squared [kgm²]) and classified according to the guidelines of the Centers for Disease Control and Prevention (underweight: ≤5th percentile; healthy weight: 5th–84th; overweight: 85th–94th; obese: ≥95th; Center for Disease Control and Prevention (34).

#### Socioeconomic status

Socioeconomic status was measured using the family’s monthly income indexed based on the social groups proposed by the Brazilian federal government protocol (25): Class A (superior to R$22,000), Class B (between R$7,000 and R$22,000), Class C (between R$3,000 and R$7,000), and Classes D/E (up to R$3,000). Parents provided the information by completing a demographic questionnaire that was sent home.

#### Daily routine, active playtime, and screen time at home

A questionnaire on children’s daily routines was used (35, 36). The questionnaire contains questions with multiple-choice answers. It is organized in five dimensions related to (1) children’s transportation from home to school (i.e., walking, car, bus, bicycle), (2) physical spaces for the child to play in during free time (i.e., parks, back yard, inside the home), (3) frequent play activities inside and outside of the house (i.e., board and tag games, coloring, drawing, music, ball games, dance) and chores at home (i.e., cleaning their room, helping with siblings, helping taking care of the house), (4) children’s interactions with other children (i.e., friends to play with from the neighborhood, school, and home backyard), and (5) administration of the children’s time using screens at home (cell phone, computer, television) and active playing outdoors during their free time (i.e., playing ball, riding a bicycle, dancing, tag games, net games). The children’s overall daily routine was used to describe the sample. Parents monitored the children’s screen time and active play outdoors daily (in minutes) for 5 consecutive days (excluding the weekends) and reported it in blocks of 30 min. Daily active play and screen time minutes were used as factors. The questionnaire has previously shown adequate internal consistency (*α* = .78) and test-retest temporal stability within a week interval (r = .83) (35).

#### Motor skill proficiency

The Test of Gross Motor Development—3rd Edition (TGMD-3 (37); validated for Brazilian children (38) was used to assess children’s MSP in locomotor skills (LOCS: run, gallop, hop, skip, horizontal jump, slide) and ball skills (BS: two-hand strike, one-hand strike, stationary dribble, catch, kick, overhand throw, underhand throw) performance. Each skill has three to five performance criteria describing the efficient movement pattern. Children were assessed following the TGMD-3 protocol recommendations contained in the manual. The motor skill proficiency trials were recorded and further analyzed. The raw score for each subtest (LOCS: 0 to 46; BS: 0 to 54) was used. Two trained professionals with previous experience with TGMD-2 and 3 conducted all the assessments and independently coded the children’s performance; both professionals coded 20% of the sample. Inter-rater reliability was high (% agreement: LOCS = 94 and BS = 96).

#### Perceived motor/athletic competence

Perceived competence (PC) was assessed using, depending on the age of the child, the physical subscale of the Pictorial Scale of Perceived Competence and Social Acceptance for children (39) and the Self-Perception Profile for Children (40). Both scales were validated for Brazilian children (16, 41), with high internal consistency (values above.80) and reliability for children 4 to 7 years old (the pictorial scale) and 8 to 12 years old (the self-profile scale). The PPC subscales have six items designed to assess children’s self-perception in the physical domain; the responses are presented on a Likert scale from one (lowest perceived competence) to four (highest perceived competence). One professional with extensive training and more than five years of experience assessing young children assessed all children individually in quiet school rooms.

#### Motor engagement with success in the lessons

Children’s appropriate motor engagement with success was assessed using a behavior checklist (16). One category of behavior was coded from the physical education lesson: Appropriate Motor Engagement with Successful (AME-S). AME-S was coded every time a child demonstrated appropriate motor engagement within the lesson and was successful in the accomplishment of the task (i.e., throw a ball at a target on the wall from a 10 m distance; toss and catch a ball without dropping it while walking on a balance beam). Four lessons were video recorded using two cameras placed in the corners for further coding of the qualitative information about children’s engagement by two coders. Two trained coders had previous experience with similar behavioral observation, independently coding all the children’s records. Coding started as the children began physical education practice. The examiner observed the children's practice for 4 min and categorized the behaviors observed. Every 4 min, another child would be observed, with information gathered from as many children as possible in one lesson. Data was recorded for all children, and each participant's mean was obtained. The intercoder reliability was high (ranging from 90% to 92%).

#### School context

The schools' physical spaces for the child to be active were examined to control the possible effects of school context on the outcomes (PA and BMI) related to the physical education courts and free play physical space (i.e., sports courts, playgrounds, wasteland) within each school. The school surroundings were also examined regarding public physical space (i.e., squares, parks, and other public spaces for physical activity), neighborhood urbanization, and demographic density. Therefore, the children from low-income families in the present study were enrolled in two different contexts. The first group—schools with more favorable physical space for the children’s movement—was composed of schools located in less urbanized neighborhoods with less demographic density; the schools also had large physical spaces for motor activities. The second group of schools—schools with less favorable physical space for the children’s movement—comprised schools located in more urbanized areas with higher demographic density and small courts for physical education lessons.

#### Procedures

The ethical committee approved the research. Administrators of six schools were contacted, and for schools that agreed to participate, meetings were held with administrators and teachers to explain the research goals and procedures. A flyer was sent home explaining the research aims and procedures; the families that agreed to participate received a consent form, a questionnaire regarding families' SES, and a questionnaire regarding children’s sociodemographic characteristics and daily routine to be completed. Classroom teachers administered the families' contact and sent and received the parental information. Parental consent and participant verbal assent were obtained for each child.

All children were assessed in the schools. The two trained professionals assessed children with MABC-2 and TGMD-3 on two alternative days; then, on the following day, the BMI and the PPC, with the help of another trained professional. PA and engagement were further assessed over the following 2 weeks during four regular physical education lessons on alternate days. For most children (above 96%), the assessments occurred in a 3-week period; for children who missed school and, thus, the assessment, the examiner team returned to the school the following week.

#### Statistical analyses

Means, standard deviations, and correlations are presented for outcomes and independent factors. Pearson and Spearman (when appropriate) correlation coefficients tests were utilized adopting the following cut-offs: *r* < .10 very weak or null; *r* = .10 to.30 as weak; *r* = .30 to.50 as moderate; *r* = .50 to.70 as moderate to strong; *r* = .70 to.90 as strong; *r* > .90 as very strong to absolute [adapted from (42)]. In order to test whether the independent variables explain significantly the outcomes (PA and BMI separately), hierarchical multiple regression was conducted. Four blocks of variables were tested. In this analysis, each block is adjusted by the prior block. Only the variables that showed a *p*-value of >.010 in the anterior block were maintained in the subsequent block. The first block included age and sex [boy or girl] as the predictors. In block two, SES [C and D classes] and School [more or less favorable space for children's movement] were also included as the predictor variable. In block three, active playtime and screen time at home were included. Finally, the model included locomotor skills, ball skills, perceived motor competence, and successful engagement in the last block. The regression models were loaded according to the children’s group.

The parameters were estimated using the least square method. The normality of residuals was examined by the Shapiro-Wilk test. In addition, a Q-Q plot with standardized residuals from the models was used to assess normality visually. Homoscedasticity was tested by examining the scatterplot of residuals. The independence of error distribution was examined using Durbin–Watson statistics. If the value is between 1.5 and 2.5, the data has no linear autocorrelation (43). Multicollinearity using the VIF (variance inflation factor) test was controlled by adopting the values above five as indicators of multicollinearity. Akaike Information Criteria (AIC) was utilized to compare the models' quality and determine which was the best fit for the data. Cohen's effect size for multiple regression (f2) was estimated adopting recognized cut-offs (f2 small < .15; f2 moderate between.15 to.34; f2 large ≥ .35) adapted from Cohen (42) and power values of ≥.80 as strong. Significance testing used an *α*-level of.05, a two-tailed test.

## Results

Table 1 presents the descriptive results by groups of children: TD, r-DCD, and DCD.

**Table 1:** Demographics by children’s groups.

Outcomes and predictors	Typical development	Risk of DCD	DCD	Total
	(*n* = 171)	(*n* = 98)	(*n* = 83)	(*n* = 352)
	F (%)	F (%)	F (%)	F (%)
Sex				
Boy	119 (69.6)	44 (44.9)	23 (27.7)	186 (52.8)
Girl	52 (30.4)	54 (55.1)	60 (72.3)	166 (47.2)
Socioeconomic status^a^				
Group C	75 (43.9)	43 (43.9)	25 (30.1)	143 (40.6)
Group D	96 (56.1)	55 (56.1)	58 (69.9)	209 (59.4)
School				
More favorable space for children's movement	103 (59.9)	60 (61.2)	55 (66.3)	281 (61.9)
Less favorable space for children's movement	68 (39.5)	38 (38.8)	28 (33.7)	134 (38.1)
	Mean (95% IC)	Mean (95% IC)	Mean (95% IC)	Mean (95% IC)
BMI (kg/m²)	17.9 (17.4–18.4)	18.4 (17.7–19.2)	19.8 (18.7–20.9)	18.5 (18.1–18.9)
Physical activity (steps per min)	72.8 (69.7–76.4)	65.8 (61.2–70.6)	60.1 (55.3–64.9)	67.9 (65.4–70.4)
Age—4 to 10 years old (year)	7.3 (7.1–7.6)	7.3 (7.0–7.7)	8.0 (7.6–8.4)	7.5 (7.4–7.7)
Active playtime at home (daily min)	192.3 (186.7–197.8)	181.3 (172.6–190.1)	190.4 (181.4–199.5)	188.7 (184.5–193)
Screen time at home (daily min)	179.0 (164.8–193.1)	179.8 (159.5–200.1)	175.5 (153.0–197.6)	178.2 (167.9–188.4)
Locomotor skills (raw score)	31.7 (30.1–34.2)	27.4 (26.4–28.4)	24.3 (23.1–25.6)	28.8 (28.1–29.4)
Ball skills (raw score)	35.5 (34.6–36.7)	28.8 (27.3–30.2)	24.6 (23.2–26.0)	31.0 (30.2–31.9)
Perceived motor competence (raw score)	19.2 (18.6–19.7)	19.1 (18.4–19.8)	17.8 (16.9–18.7)	18.8 (18.4–19.2)
Successful engagement (raw score min)	3.2 (2.8–3.6)	3.2 (2.8–3.6)	2.8 (2.3–3.4)	3.1 (2.9–3.4)

^a^ IBGE’s Brazilian indicators.

The results showed a high prevalence of overweight and obesity among all the groups of children: TD, overweight (*n* = 36; 21.1%) and obesity (*n* = 32; 18.7%); r-DCD, overweight (*n* = 13; 13.3%) and obesity (*n* = 27; 27.6%); DCD, overweight (*n* = 21; 25.3%) and obesity (*n* = 22; 26.5%). Only one TD (.6%) and one DCD child (1.2%) were categorized as underweight.

Table 2 presents the bivariate correlation of the factors with the PA and BMI outcomes by group. Several bivariate small to moderate correlations were significant for the factors and the PA outcome; less significant correlations were found for the BMI outcome.

**Table 2:** Correlation coefficient results by children’s groups.

Individual and environmental variables	Typical development (*n* = 172)				Risk of DCD (*n* = 98)				DCD (*n* = 83)			
	PA		BMI		PA		BMI		PA		BMI	
	*r*	*p*	*r*	*p*	*r*	*p*	*r*	*p*	*r*	*p*	*r*	*p*
Age (4 to 10)^a^	.423	**<** **.** **001**	.254	**.** **001**	.333	**<** **.** **001**	.301	**.** **003**	.075	.503	.236	**.** **032**
Sex [boy]^b^	.323	**<** **.** **001**	.101	.191	.225	**.** **026**	.176	.084	.183	.097	.334	**.** **002**
Socioeconomic status [D]^b^	.303	**<.001**	.037	.632	.389	**<** **.** **001**	.095	.352	.217	.049	−.021	.853
School [more favorable space for children's movement]^b^	.277	**<.001**	.161	.035	.346	**<.001**	.069	.505	.438	<.001	014	.897
Active playtime at home^a^	−.060	.432	−.106	.168	−.023	.822	−.277	.006	.079	.476	−.329	**.** **002**
Screen time at home^a^	.044	.568	.322	**<** **.** **001**	−.070	.495	.489	<.001	−.297	**.** **006**	.386	**<** **.** **001**
Locomotor skills^a^	.277	**<** **.** **001**	.031	.691	.250	.013	.194	.055	.084	.450	.102	.358
Ball skills^a^	.491	**<** **.** **001**	.130	.091	.311	.002	.228	.024	.118	.286	.230	**.** **036**
Perceived motor competence^a^	−.175	**.** **022**	−.140	.068	−.206	.042	−.321	.001	−.065	.560	−.081	.466
Successful engagement^a^	.261	**.** **001**	−.154	**.** **044**	.342	**.** **001**	−.189	.062	.479	**<** **.** **001**	−.198	.073

Correlation tests:.

^a^ Pearson.

^b^ Spearman- PA, Physical activity (steps per minute); BMI, Body mass index (kg/m^2^).

Bold values are significant at *p* < .05.

### Regression assumptions

The normality of residuals was confirmed by the Shapiro-Wilk test in all models (*p* values > .05 for all models). Q-Q plot visually confirmed the normality. The scatterplot of residuals indicated the homoscedasticity of the models. Durbin–Watson statistics indicated the independence of error distribution (values between 1.5 and 2.4). VIF test indicated no multicollinearity (values between.1 and 3.9).

### Hierarchical regression results

Table 3 presents the hierarchical regression results with the standardized regression coefficient and *p* values for the models for PA and BMI for the groups of children: DT, r-DCD, and DCD models.

**Table 3:** Hierarchical linear regression analysis with the standardized regression coefficient (b) and significant results (*p*).

Individual and environmental variables		Typical develop (*n* = 172)				Risk of DCD (*n* = 98)				DCD (*n* = 83)			
		PA		BMI		PA		BMI		PA		BMI	
		*b*	*p*	*b*	*p*	*b*	*p*	*b*	*p*	*b*	*p*	*b*	*p*
Model 1	Age (4 to 10 years)	.380	**<** **.** **001**	.246	.001	.313	**.** **002**	.288	.004	.126	.253	.292	.005
	Sex [boy]	.299	**<** **.** **001**	.076	.314	.212	**.** **029**	.179	.070	.231	**.** **038**	.377	<.001
Model 2	SES [class D]	.260	**<** **.** **001**	−.037	.668	.417	**<** **.** **001**	.030	.781	.213	**.** **028**	−.172	.122
	School [more favorable space for children's movement]	.234	**<** **.** **001**	−.102	.239	.394	**<** **.** **001**	.010	.924	.465	**<** **.** **001**	.057	.618
Model 3	Active playtime at home	.022	.737	−.050	.497	.113	.183	−.284	**.** **003**	.085	.374	−.229	**.** **027**
	Screen time at home	−.015	.814	.251	**.** **001**	.030	.719	.255	**.** **006**	−.223	**.** **024**	.219	**.** **028**
Model 4	Locomotor skills	.170	**.** **029**	−.182	**.** **039**	.210	**.** **024**	−.101	.511	.006	.995	.127	.259
	Ball skills	.272	**.** **009**	−.099	.360	−.115	.227	−0.079	.692	.094	.399	−.031	.792
	Perceived motor competence	−.064	.351	.009	.905	−.031	.736	−.138	.186	.107	.273	−.119	.248
	Successful engagement	.186	**.** **003**	−.176	.**015**	.153	.069	−.141	.137	.304	**.** **002**	−.126	.210

Reference category between [ ]- PA, Physical activity (steps per minute); BMI, Body mass index; *p*, significant values.

Bold values are significant at *p* < .05.

Table 4 presents the R squared, F squared, and power observed in each block from hierarchical regression models, according to the group. In general, moderate to large effect sizes and high power for the models of children with TD, r-DCD, and DCD were observed.

**Table 4:** R squared, F squared, and test power for each hierarchical regression model block by children’s group.

Individual and environmental variables		Typical development (*n* = 172)						Risk of DCD (*n* = 98)						DCD (*n* = 83)					
		PA			BMI			PA			BMI			PA			BMI		
		*R* ^2^	*f^2^*	*P*	*R* ^2^	*f^2^*	*P*	*R* ^2^	*f^2^*	*P*	*R* ^2^	*f^2^*	*P*	*R^2^*	*f^2^*	*P*	*R* ^2^	*f^2^*	*P*
Model 1	Age (4 to 10 years)	.13	.15	.99	.06	.06	.82	.13	.15	.93	.13	.15	.93	.04	.04	.34	.17	.20	.95
	Sex [boy]																		
Model 2	SES [class D]	.31	.45	1.0	.06	.06	.82	.35	.54	.99	.35	.54	.99	.26	.35	.99	.18	.22	.97
	School [more favorable space for children's movement]																		
Model 3	Active playtime at home	.30	.43	1.0	.12	.14	.99	.35	.54	.99	.22	.28	.99	.30	.42	.99	.27	.37	.99
	Screen time																		
Model 4	Locomotor skills	.37	.59	.99	.15	.18	.99	.39	.64	.99	.23	.30	.99	.37	.59	.99	.25	.33	.99
	Ball skills																		
	Perceived motor competence																		
	Successful engagement																		

Cohen *f*^2^ effect size: *f*^2^ small < .15- *f*^2^ moderate from .15 to .34- *f*^2^ large > .35- P = power, P strong > .80. PA, Physical activity; BMI, Body mass index; DCD, Developmental coordination disorder.

### Children with typical development

The first model was significant [*F*(2,170) = 7.947, *p* = .001, adjusted *r*^2^ = .13, *f*² = .15], and both age (*b* = .380, *p* < .001) and sex [boy] (*b* = .299, *p* = .002) positively explained the PA with a moderate effect size. The second model was also significant [*F*(4,168) = 19.907, *p* < .001, adjusted *r*^2^ = .31, *f*² = .45], with a large effect size; SES (*b* = .260, *p* = .001) and school [More opportunities for movement] positively explained this outcome (*b* = .234, *p* = .002). Model 3 was also significant [*F*(6,163) = 13.159, *p* < .001, adjusted *r*^2^ = .30, *f*² = .43], with a large effect size, but active playtime and screen time at home were non-significantly associated with PA. The last model [*F*(7,165) = 7.947, *p* = .001, adjusted *r*^2^ = .37, *f*² = .59] showed that locomotor skills (*b* = .170, *p* = .029), ball skills (*b* = .272, *p* = .009), and successful engagement (*b* = .186, *p* = .003) significantly and positively explained the PA, with a large effect size. This last model showed the lowest AIC (value = 1,506).

Concerning BMI outcome, in the first model [*F*(2, 170) = 6.312, *p* = .002, adjusted *r*^2^ = .06, *f*² = .06], with a small effect size, only age was significantly and positively associated with this outcome (*b* = .246, *p* = .001). In the second model [*F*(4,168) = 5.492, *p* = .001, adjusted *r*^2^ = .06, *f*² = .06], with a small effect size). However, neither SES [D class] or more opportunities for movement in the School was significant. In the third model [*F*(3,169) = 8.409, *p* < .001, adjusted *r*^2^ = .12, *f*² = .14, with a small effect size], screen time (*b* = .251, *p* = .027) was positively associated with BMI. The last model [*F*(6,166) = 6.088, *p* < .001, adjusted *r*^2^ = .15, *f*² = .18, with a moderate effect size] showed that locomotor skills (*b* = −.182, *p* = .039) and successful engagement (b = -.176, *p* = .015) significantly and negatively explained the BMI. A lower AIC (value = 853) was observed in model 4.

### Children at risk of developmental coordination disorder

Concerning the PA outcome, the analysis showed that the first model was significant [*F*(2,96) = 7.947, *p* = .001, adjusted *r*^2^ = .13, *f*² = .15, with a moderate effect size]. Age and sex [boy] factors were significantly and positively associated with PA (*b* = .313, *p* = .002 and *b* = .212, *p* = .029, respectively). The second model was significant [*F*(4,94) = 13.667, *p* < .001, adjusted *r*^2^ = .35, *f*² = .54, with a large effect size]. In this model, SES [D class] (*b* = .417, *p* < .001) and School [More opportunities for movement] (*b* = .394, *p* < .001) positively explained the PA. Model 3 was also significant [*F*(5,93) = 11.128, *p* < .001, adjusted *r*^2^ = .35, *f*² = .54], with a large effect size. Nevertheless, active playtime and screen time at home were non-significantly associated with PA. The fourth model was significant [*F*(7,91) = 9.674, *p* < .001, adjusted *r*^2^ = .39, *f*² = .64], with a large effect size. Only locomotor skill was significantly associated with PA (*b* = .210, *p* = .024). The second model showed the lowest AIC (value = 825).

Regarding the models with BMI outcomes, the first model [*F*(2,96) = 7.947, *p* = .001, adjusted *r*^2^ = .13, *f*² = .15, with a moderate effect size] showed that only age was significantly and positively associated with this outcome (*b* = .288, *p* = .004). The second model was significant [*F*(3,95) = 2.965, *p* < .036, adjusted *r*^2^ = .35, *f*² = .54, with a large effect size]; however, SES and School [More opportunities for movement] were non-significantly associated with BMI. The third model was significant [*F*(3,95) = 9.917, *p* < .001, adjusted *r*^2^ = .22, *f*² = .28, with a moderate effect size]; active playtime at home negatively explained BMI (*b* = -.284, *p* = .003), while screen time positively explained this outcome (*b* = .255, *p* = .006). The last model was also significant [*F*(7,91) = 5.007, *p* < .001, adjusted *r*^2^ = .230, *f*² = .30, with a moderate effect size]; however, none of the variables included in this block were significantly associated with BMI. Model 3 showed the lowest AIC (value = 511).

### Children with developmental coordination disorder

For the PA outcome, the results showed that the first model was significant [*F*(2,81) = 2.589, *p* = .049, adjusted *r*^2^ = .04, *f*² = .04], with a small effect size. Only sex [boy] was significantly and positively associated with PA (*b* = .231, *p* = .038). The second model was also significant [*F*(3,80) = 10.689, *p* < .001, adjusted *r*^2^ = .26, *f*² = .35], with a large effect size; SES [D class] and school [More opportunities for movement] were significantly and positively associated with PA (*b* = .213, *p* = .028 and *b* = .465, *p* < .001, respectively). The third model was also significant [*F*(5,78) = 8.201, *p* < .001, adjusted *r*^2^ = .30, *f*² = .42], with a large effect size; screen time was significantly and negatively associated with PA (*b* = −.223, *p* = .024). Model 4 also was significant [*F*(8,75) = 7.013, *p* < .001, adjusted *r*^2^ = .37, *f*² = .59], with a large effect size; successful engagement was significantly and positively associated with PA (b = .304, *p* = .002). The lowest AIC (value = 723) was observed in model 4.

Regarding BMI outcome, the first model was significant [*F*(2, 81) = 9.695, *p* < .001, adjusted *r*^2^ = .17, *f*² = .20], with a moderate effect size; age and sex [boy] were significant and positively associated with BMI (*b* = .292, *p* = .005 and *b* = .377, *p* < .001, respectively). The second model was significant [*F*(4,79) = 5.492, *p* = .001, adjusted *r*^2^ = .18, *f*² = .22], with a moderate effect size; neither SES [D class] nor School (*b* = .213, *p* = .028) were significant. The third model was significant [*F*(4,79) = 8.501, *p* < .001, adjusted *r*^2^ = .27, *f*² = .37], with a large effect size; active playtime at home (*b* = −.229, *p* = .027; negative association) and screen time at home (*b* = .219, *p* = .028; [positive association) were significantly associated with BMI. The last model also was significant [*F*(7,76) = 4.873, *p* < .001, adjusted *r*^2^ = .25, *f*² = .33], with a moderate effect size; no predictor in this block was significantly associated with BMI. A lower AIC (value = 480) was observed in model 3.

## Discussion

Regarding DCD prevalence, the results showed that 23.6% of children had DCD and 27.8% were at risk of DCD, like previous studies in Brazil. To illustrate, for children from low-income families, two previous studies in the South of Brazil showed a DCD prevalence of approximately 20%, and 17% were found to be at risk of DCD (44, 30). In Southwest Brazil, 30% of children were reported to be at risk of DCD, with a prevalence three times higher in low-income families (48%) than in middle-class families (15%) from São Paulo (45), and in Minas Gerais, the prevalence of DCD was approximately 23% [Cardoso & Magalhães (46)]. In the Northeast of Brazil, a DCD prevalence of 47% an 11.6% was reported in Paraiba (47), and in the state of Ceará, respectively. Also, 25.8% of children were detected with of risk at DCD in state of Ceará (48). In the north of Brazil, for Manaus, the prevalence of DCD was 45% in one study (49) and in another, 33% (50). Most of these studies have children’s social and economic vulnerability in common. For example, in one study, SES explained 23% of the variance in motor percentile scores, robustly suggesting that low SES enhances the risk of poor motor development (30). In the present study, all children were from low-income families, and 59.4% lived at or below the poverty line; therefore, the prevalence could be influenced by a combination of economic and contextual factors, endangering children’s health and opportunities for development.

This study is the first to examine how SES, school characteristics related to the physical space for the children to move, active playtime, screen time at home, MSP (locomotor and ball skills), perceived competence, and appropriate motor engagement with success in physical education lessons were associated with physical activity and BMI in children from low-income families with typical development, at risk of DCD, and with DCD (r-DCD). In this study, we examined those associations in a large sample with an extensive age range of 4 to 10 years old.

The hierarchical regression analysis showed that sex was associated with PA for children with TD, r-DCD, and DCD (b values above 20) with moderate to large effect sizes. The results showed that boys were more active than girls in the lessons across the three groups; similar results were previously reported. Boys spent significantly more time in moderate and vigorous physical activity than girls during physical education lessons (51, 31). Furthermore, boys tended to be more active throughout the day; two systematic reviews of 48 studies confirm this trend (52, 10). A probable explanation for those results was that boys view physical education lessons as an opportunity to be active despite the lesson’s content, whereas girls need further encouragement from teachers and peers to be engaged. The results show that there is a need to establish why boys are more attracted to any activity and consequently become more physically active than girls during physical education lessons; this can be considered a recommendation for future research, as it could inform strategies to promote physical activity for girls.

The analysis also showed that lower SES (class D) and schools with more opportunities for movement significantly explained the PA in all groups of children with high effect sizes. On the contrary, these variables did not explain BMI. Interestingly, although all children were from low-income families, the findings indicate that children in the line of poverty (group D) were more active in the lessons. For children living in poverty, the most regular PA occurs informally at home or around the neighborhood (53, 54). For children in this study, physical education lessons are one of the few opportunities to engage in organized activities with different types of equipment; they probably try to get the most out of it.

Children from low-income families have no means to join sports clubs or even pay for children’s mobility to other neighborhoods to attend sports programs as, often, sports clubs are not located in poor, peripheric regions. Thus, opportunities for oriented motor practices in physical education lessons and school physical spaces to play with friends (i.e., sports courts, playgrounds, wastelands) were positively associated with PA, independently of the children’s range of motor proficiency (TD, r-DCD, and DCD) of children. These factors act like a compensatory force for increasing the PA in TD, r-DCD, and DCD children, even though they are from low-income families. These findings emphasize the need to reinforce and redesign the school’s role in physical activity behavior and its possible benefit to children from low-income families. It is of note that the school’s context factor (schools with available physical space for child move) was not relevant for the BMI models. BMI is highly associated with families consuming food containing added sugar, and children are more likely to have no control over these specific matters (55); food intake was not investigated in the present study, which is a limitation. Furthermore, although the school surroundings have space for children to move, parents may not feel safe allowing children to play outside due to safety concerns (56). Consequently, it is plausible to assume that in this context, more physical space to play outside the home had little relation to children’s BMI.

Two measures of the child’s daily routine at home were also assessed: screen time and active playtime at home. Both were significant in the BMI models for children with r-DCD and DCD, with moderate to large effect sizes; screen time was also significant in BMI for children with TD. Further, screen time was negatively associated with PA in physical education lessons in DCD children. Children with higher BMI were the ones who spent more time using screens and less time in active play (r-DCE and DCD) at home. Robust relationships between screen time and weight status have also been reported in Greek children (14) and Australian adolescents (57), corroborating the results of this study. These results prove that children with movement difficulties are less vigorously and physically active at home and engage more in solitary activities such as watching TV, which is the primary screen time in the present study; they are likely to spend less time in peer social interactions, losing opportunities to learn new skills.

The results showed that LOCS and BS were positively and significantly associated with PA only for children with TD, and the model showed a large effect size; LOCS were positively associated with PA in r-DCD, with a large effect size; no associations were found for children with DCD. The research evidence for the relationship between MC and different levels of PA in typical children has been robust in the literature for the last decades (12), mainly BS, since proficiency during childhood impacts future physical activity (6). Concerning the lack of associations between MSP status and PA for children with DCD, a plausible explanation is that the children’s motor impairments lead to more frequent failures in the tasks. Engagement with success means was lower for children with DCD, restraining those children from being effective in PA during the lessons and the steps per minute were also lower for children with DCD. Several studies support the outcome of the present study by providing undoubted evidence that children’s motor skill levels were related to the levels of physical activity of those children. For example, Vandorpe et al. (58) reported that children who were less coordinated were consistently less engaged in sporting activities over the three years due to difficulties in learning new skills.

In this study, LOCS was negatively associated with BMI in children with TD; the model showed a large effect size. Previous studies reported an association between locomotor proficiency and BMI in typically developing children (59); (Ramirez, Pérez-Cañaveras Herrero, 2021); (60, 61, 62, 63, 64). However, the extensive literature evidence indicates the BMI effect in LOCS (65, 66, 61, 64) or a significant but non-directional relationship (i.e., correlation) (62); (67) (68); between these variables. Few studies have investigated the effect of locomotor skills on BMI (59, 69). For example, Webster et al. (59) investigated the relationship between fundamental motor skills (LOC and BS) and BMI in TD children, like the present study, and they also observed that locomotor skills were significantly and negatively associated with BMI.

The present study’s findings provide evidence that locomotor skills performance is a potential protective factor for overweight and obesity in children with TD. However, it is vital to note that this relationship was not observed for children with DCD and at r-DCD. In those groups of children, locomotor proficiency was lower than in TD children. It seems reasonable to assume that a more comprehensive range of motor proficiency is necessary to detect this relationship. More studies are needed to address this question appropriately, especially among children with DCD and r-DCD.

Concerning the successful engagement results, the analysis showed that TD and DCD children who completed the task also showed more PA during physical education lessons. For children with TD, it was also inversely related to BMI: children with lower BMI in the TD groups were also the ones with more experience of success during the practice of motor tasks. The results aligned with a previous study involving older TD children and showed a positive relationship between appropriate engagement with PA (16). However, here, we expand on previous knowledge by showing a similar trend for young TD and DCD children and showing that children’s success during the lessons is crucial. Children, even those with DCD, who experience repeated task successes are likely to experience a heightened sense of efficacy and persistence, whereas those who encounter difficulties are inclined to remain inefficacious and give up on the challenge. Furthermore, once a strong sense of efficacy is embedded, occasional failures should not affect children’s behavior; failure could even lead to sustained effort, resulting in success, which implies that obstacles could be overcome (70). The results suggested that repeated opportunities to practice with success may help consolidate high PA levels, which are crucial for children to acquire the daily threshold of at least 60 min of moderate to vigorous physical activity (1), and they may, in the long run, transfer this behavior to new experiences. Investigating whether experience of success predicts PA and BMI is a recommendation for further studies.

Furthermore, early evidence suggested that children with DCD were less vigorously physically active during recess at school (71). It seems that they are less likely to be physically active due to a fragile sense of self-efficacy (72), and the desire to withdraw from the activities may also be reinforced by peers, which is a strong possibility during physical education lessons (73). Our results reinforce the positive effect of successful engagement in PA in physical education lessons, even in children with DCD, who have more difficulty being physically active.

The results of the current study expand on previous knowledge by addressing children’s experiences of success in lessons, screen time, and active playtime at home in relation to PA and BMI. We found that the more frequent the experiences of success, the higher the levels of PA for all children, and the higher the screen time, the higher the BMI, regardless of group. The results also showed that children with r-DCD and DCD with high BMI engage less in active play at home.

## Limitations and strengths

This study has several limitations. First, as pedometers were used instead of accelerometers, the intensity of physical activity could not be measured. Second, knowing why girls engage in some tasks and are requested to partake in others could provide insights into the different behaviors of boys and girls in physical education lessons. However, it was not assessed in the present study. Third, girls seem to rely more upon teachers' and peers' encouragement during the lessons; although this was observed in the video records, it was not the study’s goal and, therefore, it was not assessed. Third, parents monitored the children’s screen time and active playtime daily for five consecutive weekdays and reported it in blocks of 30 min; the duration of screen time was assessed. Although self-reporting by proxy measures of screen use and duration time are the prevalent methods of assessing screen time (74), objective measures such as mobile devices could be more accurate in assessing screen time and active playtime in children. Fourth, children's food intake habits were not measured, and this factor needs to be considered in studies addressing BMI as an outcome in the models.

This study has several strengths. This study expands on previous knowledge by investigating individual and environmental factors associated with PA and BMI for children with different levels of motor proficiency: children with r-DCD, DCD, and TD. An additional strength of this study resides in the comprehensive investigation of a combination of multiple factors, including extensively investigated factors such as motor proficiency and perceived athletic/motor competence but also factors that are often overlooked in the literature: schools' physical spaces that are favorable or not for children’s movement experiences, the quality of children's engagement in physical education lessons, active playtime at home, and screen time at home. This approach has afforded us an enhanced grasp of the relationship between these variables within the distinct motor impairment levels of the children under examination.

## Conclusions

Since PA and BMI are recognized as constructs critical for children's participation in health activities and sports, due to their relevance, it is necessary to ensure all children have adequate practice opportunities, regardless of their levels of MC. Overall, from the results of the present study, age, sex, SES, school physical space favorable to children’s movement, active playtime at home, screen time at home, locomotor and ball skills, and engagement in physical education were associated with PA and BMI for children with different levels of motor competence. Some of these factors are, to some extent, in the control of the educators. Therefore, educators should foster the learning of motor skills and promote experiences of successful engagement in lessons despite the levels of children's MC.

Furthermore, SES was also associated in the present study with PA. It is essential to develop strategies to engage socioeconomically disadvantaged children in PA as schools may be the only opportunity for those children to be active. By setting challenging and achievable goals for each child within the school program and building on the children’s desires and needs, physical education teachers may be able to help children meet the PA guidelines to be healthy. Furthermore, in the present study, screen time was above the recommended time for children, at almost 3 h daily; educating parents about the importance of active playtime may be a straightforward way to decrease screen time among children.

## Data Availability

The raw data supporting the conclusions of this article will be made available by the authors, without undue reservation.
